# Clinically mild encephalitis/encephalopathy with a reversible splenial lesion caused by methicillin-sensitive *Staphylococcus aureus* bacteremia with toxic shock syndrome: a case report

**DOI:** 10.1186/s12879-016-1516-0

**Published:** 2016-04-18

**Authors:** Koki Kosami, Tsuneaki Kenzaka, Yuka Sagara, Kensuke Minami, Masami Matsumura

**Affiliations:** Division of General Internal Medicine, Jichi Medical University Hospital, Shimotsuke, Japan; Division of Community Medicine and Career Development, Kobe University Graduate School of Medicine, 2-1-5, Arata-cho, Hyogo-ku, Kobe, Hyogo 652-0032 Japan; Department of Breast Surgery, Jichi Medical University, Shimotsuke, Japan; Department of General Medicine, Toyooka Public Hospital, Toyooka, Japan

**Keywords:** Clinically mild encephalitis/encephalopathy with a reversible splenial lesion, Toxic shock syndrome, *Staphylococcus aureus*, Bacteremia

## Abstract

**Background:**

Clinically mild encephalitis/encephalopathy with a reversible splenial lesion (MERS) is a mild encephalopathy caused by various pathological processes, but encephalopathy due to bacteria is rare.

**Case presentation:**

We report the case of a 45-year-old Japanese woman who on receiving chemotherapy for advanced breast cancer developed an altered mental status and dysarthria soon after fever from infection of a subcutaneous implantable port. *Staphylococcus aureus* was detected in her blood cultures. Magnetic resonance imaging (MRI) revealed an ovoid lesion in the central portion of the splenium of the corpus callosum (SCC). Although hypotension was not observed, we diagnosed probable toxic shock syndrome (TSS) based on fever (temperature: >38.9 °C), altered mental status, erythema, desquamation, thrombocytopenia, liver dysfunction, and creatine phosphokinase elevation. We administered antimicrobial therapy and her neurological symptoms improved gradually. The lesion in the SCC completely disappeared on MRI 7 days after disease onset.

**Conclusions:**

We diagnosed this case as MERS caused by *S. aureus* bacteremia with TSS. This is the first report of such a case, and we suggest that when a TSS patient presents with neurological symptoms, the possibility of MERS should be considered.

## Background

Mild encephalitis/encephalopathy with a reversible splenial lesion (MERS) is a mild encephalopathy caused by various pathological processes such as viral or bacterial infections, drugs, metabolic and electrolyte abnormalities, and trauma [1]. It is characterized by neurological symptoms such as consciousness, vertigo, and seizure.

Several viruses (influenza virus, adenovirus, mumps virus, varicella zoster virus [2, 3], rotavirus [4], measles virus [5], and hepatitis A virus [6]) and bacteria (*Escherichia coli* [7], *Legionella pneumophila* [8], and *Mycoplasma pneumoniae* [9]) have been reported to cause MERS, but bacteria are rarely the cause [2, 3]. *Staphylococcus aureus*, especially, is a very rare cause of MERS, and only one such case has been reported so far [10]. Moreover, there are no reports on MERS associated with toxic shock syndrome (TSS).

We encountered a case of MERS caused by *S. aureus* bacteremia with TSS and report our findings in this study.

## Case presentation

A 45-year-old Japanese woman had undergone chemotherapy for advanced breast cancer (TNM Classification for Breast Cancer, T4N0M0; clinical Stage IIIb) for 2 years. She had a subcutaneous implantable port attached to her left subclavian vein and was receiving vinorelbine 11 days before admission. A second dose of vinorelbine was administered 4 days before admission. She developed fever following pain and swelling at the site of the implantable port 2 days before admission. She was suspected to have catheter-related bloodstream infection and thus admitted to our hospital. The implantable port was removed and piperacillin/tazobactam was administered at 4.5 g every 8 h. On hospital day 2, altered mental status and dysarthria were observed.

**Fig. 1:**
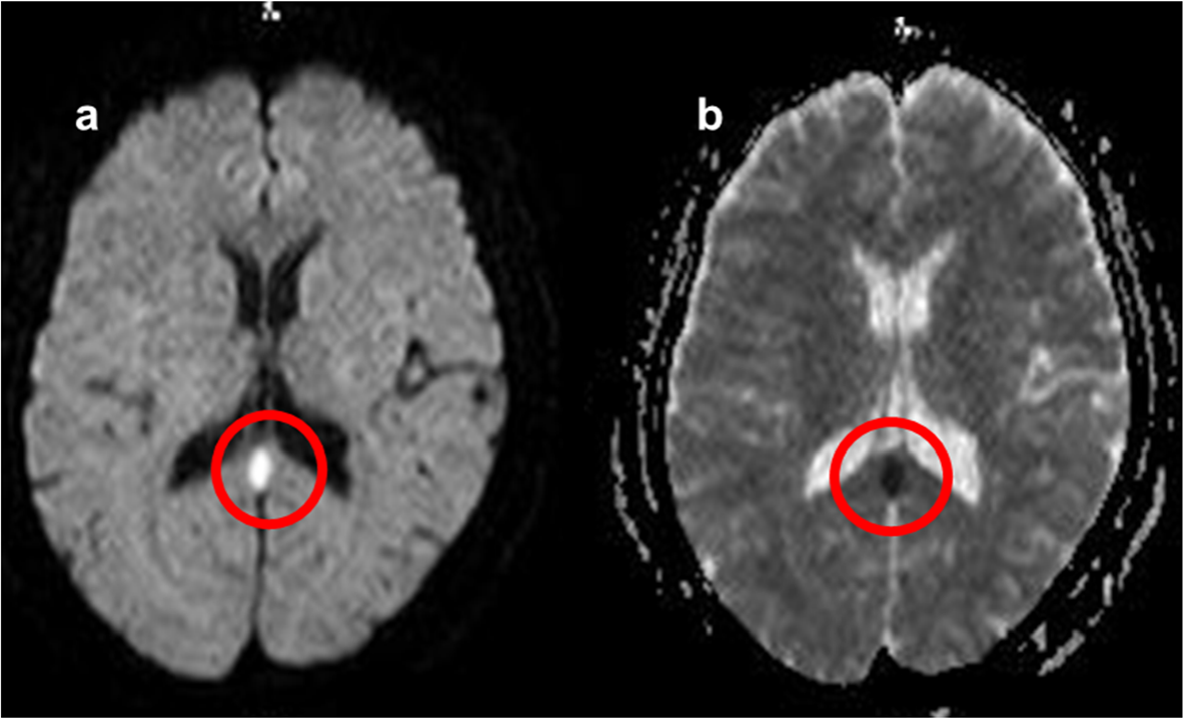
Head magnetic resonance imaging on admission day 2. **a** Diffusion-weighted imaging (DWI), **b** Apparent diffusion coefficient (ADC). Head magnetic resonance imaging revealed an ovoid lesion in the central portion of the splenium of the corpus callosum. The lesion appears as reduced diffusion on DWI and has a low ADC value (red circles)

At the onset of neurological symptoms, her vital signs were as follows: blood pressure, 109/63 mmHg; pulse, 96 bpm; respiratory rate, 26 bpm; and temperature, 39.5 °C. The Glasgow Coma Scale score for consciousness was 13 (eye opening, 4; verbal response, 4; and best motor response, 5). No obvious paralysis was noted, although evaluation of neurological symptoms was difficult because of disorders of consciousness. Conjunctival hyperemia was noted. Except for the subclavian lesion at the site of the implantable port, no abnormalities of the skin, lung, heart, and abdomen were noted. The subclavian lesion was red and swollen. Laboratory test results were as follows: white blood cell count 400/mm^3^; hemoglobin, 9.9 g/dl; platelet, 6.2 × 10^4^/mm^3^; blood urea nitrogen, 15 mg/dl; creatinine, 0.69 mg/dl; aspartate aminotransferase, 48 U/l; alanine aminotransferase, 26 U/l; lactate dehydrogenase, 353 U/l; creatine phosphokinase, 1859 U/l; serum sodium, 130 mEq/l; serum potassium, 3.3 mEq/l; and C-reactive protein, 13.5 mg/dl (Table 1). Head magnetic resonance imaging (MRI) revealed an ovoid high-intensity lesion in the central portion of the SCC on diffusion-weighted imaging (DWI, Fig. 1). Based on the MRI findings, septic embolus or cerebral infarction was considered because the neurological symptoms occurred suddenly following fever due to catheter-related bloodstream infection. On the same day, gram-positive cocci were detected in blood cultures. Therefore, on hospital day 2, piperacillin/tazobactam therapy was switched to cefepime and vancomycin. On hospital day 4, methicillin-sensitive *S. aureus* (MSSA) was detected in blood cultures. Nafcillin and oxacillin, the first treatment of choice for MSSA-related diseases of the central nervous system, are not available in Japan. Therefore, cefepime was administered at 2 g every 8 h.

**Table 1:** Laboratory data on hospital day 2

Parameter	Recorded value	Standard value
White blood cell count	0.40 × 10^9^/L	4.00–7.50 × 10^9^/L
Neutrophil	0.30 × 10^9^/L	
Monocyte	0.01 × 10^9^/L	
Lymphocyte	0.09 × 10^9^/L	
Hemoglobin	9.9 g/dL	11.3–15.2 g/dL
Hematocrit	35.8 %	36–45 %
Platelet	6.2 × 10^9^/L	130–350 × 10^9^/L
C-reactive protein	13.5 mg/dL	≤0.14 mg/dL
Total protein	6.1 g/dL	6.9–8.4 g/dL
Albumin	2.9 g/dL	3.9–5.1 g/dL
Aspartate aminotransferase	48 U/L	11–30 U/L
Alanine aminotransferase	26 U/L	4–30 U/L
Lactate dehydrogenase	353 U/L	109–216 U/L
Creatine phosphokinase	1859 U/L	40–150 U/L
Blood nitrogen urea	15 mg/dL	8–20 mg/dL
Creatinine	0.69 mg/dL	0.63–1.03 mg/dL
Sodium	138 mEq/L	136–148 mEq/L
Potassium	4.2 mEq/L	3.6–5.0 mEq/L
Glucose	124 mg/dl	70–109 mg/dl

**Fig. 2:**
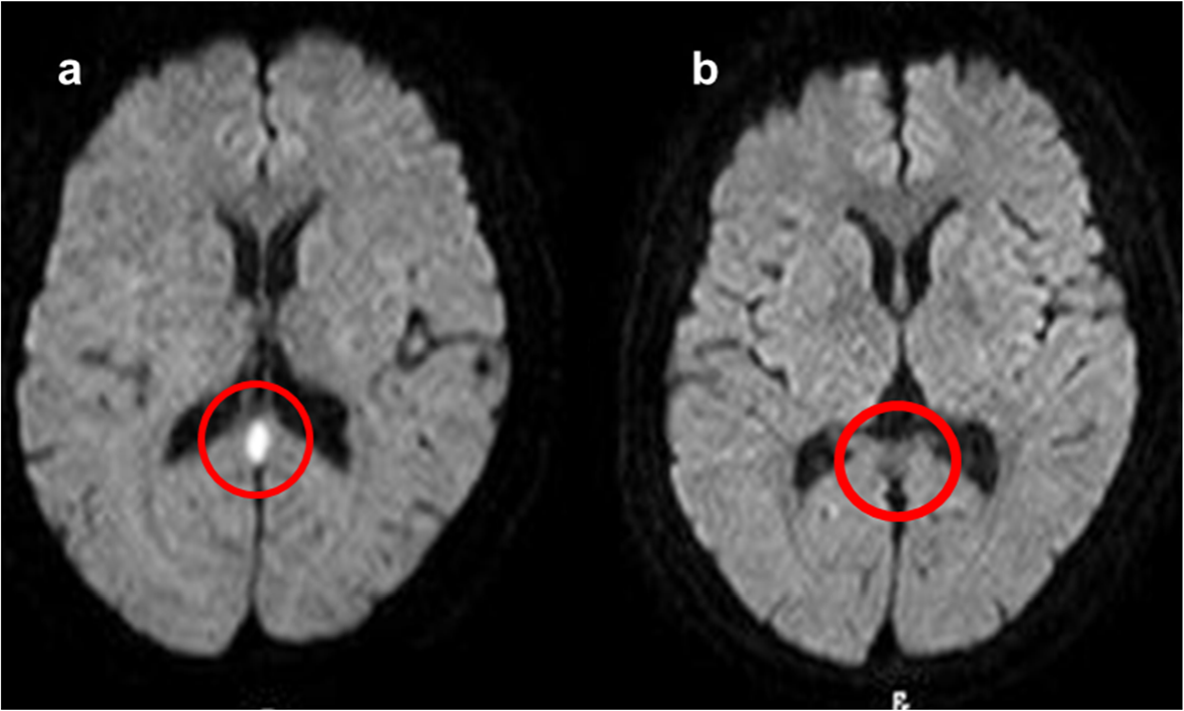
Head magnetic resonance imaging (MRI). **a** Diffusion-weighted imaging on admission day 2, **b** Diffusion-weighted imaging on admission day 9. Abnormalities detected on MRI completely disappeared on admission day 9 (red circles)

The patient's altered mental status and dysarthria improved gradually. On hospital day 9, 7 days after disease onset, the ovoid lesion had completely disappeared on MRI (Fig. 2).

Conjunctival congestion and erythema developed on hospital days 2 and 3, respectively. Aspartate aminotransferase, alanine aminotransferase, and creatine phosphokinase levels were high at 137, 88, and 11,240 U/l on hospital days 8, 4, and 8, respectively. Soon erythema developed, and desquamation occurred 2 weeks after disease onset. In addition, isolated MSSA strains produced toxic shock syndrome toxin-1 (TSST-1). Although hypotension was not seen, probable TSS was diagnosed from fever (temperature, >38.9 °C), erythema, desquamation, creatine phosphokinase elevation, liver dysfunction, thrombocytopenia, and altered mental status [11, 12].

On hospital day 12, we switched from cefepime to vancomycin plus ceftriaxone because of drug eruption. On hospital day 15, we switched from vancomycin plus ceftriaxone to cefazolin at 2 g every 8 h, because due to the following reasons we concluded it was not necessary to consider cerebrospinal fluid (CSF) penetration by the antimicrobial agent: (i) CSF examination results were unremarkable and culture negative, (ii) abnormal findings on head MRI were due to MERS, and (iii) multiple organ injuries were due to TSS.

**Fig. 3:**
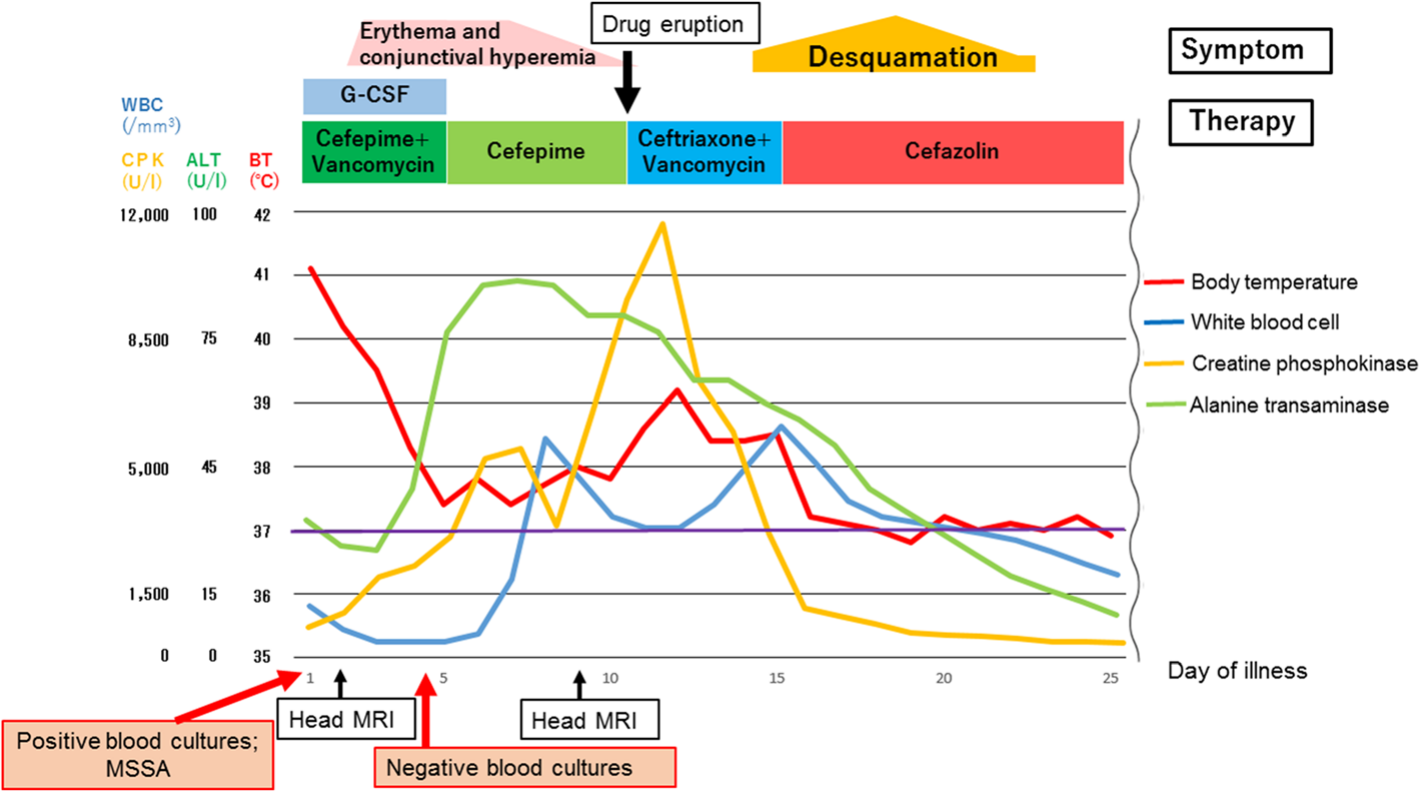
Patient's clinical course after hospitalization. Abbreviations: MSSA, methicillin-sensitive *Staphylococcus aureus*; magnetic resonance imaging, MRI

Figure 3 shows the clinical course post admission. Transesophageal echocardiography revealed no abnormalities. Moreover, septic emboli were not detected by enhanced computed tomography of the neck, chest, abdomen, and pelvis. We continued antibiotic therapy for 4 weeks after the blood cultures tested negative, i.e., after hospital day 4. At the 6-month follow up, no recurrence was noted.

## Discussion

We describe a rare case of MERS caused by *S. aureus* bacteremia with probable TSS. MERS is caused by various viruses, such as influenza, mumps, adenovirus, varicella, and zoster virus, but rarely by bacteria [7, 9]. Thus far, only one case of MERS caused by *S. aureus* has been reported [10]. To the best of our knowledge, this is the first reported case of MERS with TSS.

MRI is useful for diagnosing MERS [1, 13] as MRI scans can reveal lesions in the SCC. In a case reported by Doherty et al., the lesion presented as reduced diffusion on DWI, had a low ADC value, and disappeared within a month. The radiological findings of our case were typical of MERS [1]. Acute disseminated encephalomyelitis (ADEM) is suggested to be considered as a differential diagnosis of postinfection encephalopathy [13]. In our case, the splenial lesion completely disappeared a week before clinical recovery, thus ruling out ADEM. Other differential diagnoses include ischemia, posterior reversible encephalopathy syndrome, diffuse axonal injury, multiple sclerosis, hydrocephalus, Marchiafava-Bignami disease, lymphoma, and extrapontine myelinolysis [13]. All were excluded clinically.

Our patient had a catheter-related bloodstream infection caused by MSSA. Probable TSS was diagnosed as the case met all the diagnostic criteria except hypotension and gastrointestinal symptoms [12] (Table 2). TSS is characterized by multiple organ impairment due to *S. aureus* exotoxin [11]. Staphylococcal exotoxins can activate several T cells at once, resulting in massive cytokine production [14]. Activated T cells release interleukin (IL)-1, IL-2, tumor necrosis factor (TNF)-alpha, TNF-beta, and interferon-gamma in large amounts, resulting in TSS [15]. Although the mechanism of MERS is unknown, Tada et al. postulated two possible mechanisms for the transiently decreased ADC value of the lesions, intramyelinic edema, and inflammatory infiltrate [2]. We suppose that the inflammatory infiltrate caused by cytokine cascades of TSS are involved in the pathogenesis of MERS in our case.

**Table 2:** Diagnostic categories of toxic shock syndrome (TSS)

Definite TSS (all criteria must be present) [11]	
Clinical Criteria	
An illness with the following clinical manifestations:	
• Fever: temperature greater than or equal to 102.0 °F (greater than or equal to 38.9 °C)	
• Rash: diffuse macular erythroderma	
• Desquamation: 1–2weeks after onset of rash	
• Hypotension: systolic blood pressure less than or equal to 90 mmHg for adults or less than the fifth percentile by age for children aged less than 16 years	
• Multisystem involvement (three or more of the following organ systems):	
º Gastrointestinal: vomiting or diarrhea at onset of illness	
º Muscular: severe myalgia or creatine phosphokinase level at least twice the upper limit of normal	
º Mucous membrane: vaginal, oropharyngeal, or conjunctival hyperemia	
º Renal: blood urea nitrogen or creatinine at least twice the upper limit of normal for laboratory or urinary sediment with pyuria (greater than or equal to 5 leukocytes per high-power field) in the absence of urinary tract infection	
º Hepatic: total bilirubin, alanine aminotransferase enzyme, or aspartate aminotransferase enzyme levels at least twice the upper limit of normal for laboratory	
º Hematologic: platelets less than 100,000/mm^3^	
º Central nervous system: disorientation or alterations in consciousness without focal neurologic signs when fever and hypotension are absent	
Laboratory Criteria for Diagnosis	
Negative results on the following tests, if obtained:	
• Blood or cerebrospinal fluid cultures (blood culture may be positive for *Staphylococcus aureus*)	
• Negative serologies for Rocky Mountain spotted fever, leptospirosis, or measles	
Probable TSS (≥3 criteria and desquamation or ≥5 criteria without desquamation) [12]	
• Temperature ≥38.9 °C	
• Rash	
• Hypotension, orthostatic dizziness, or syncope	
• Myalgia	
• Vomiting, diarrhea, or both	
• Mucous membrane inflammation (conjunctivitis, pharyngitis, vaginitis)	
• Clinical or laboratory abnormalities of ≥2 organ systems	
• Reasonable evidence for absence of other etiologies	

Although MERS coexisting with TSS is extremely rare, staphylococcal exotoxins may cause MERS. Since our patient did not have hypotension, we could perform head MRI. Typically, TSS presents with hypotension and poor general conditions, hindering MRI. Thus, one cannot ignore MERS coexisting with TSS.

## Conclusions

To conclude, we report the world's first case of MERS caused by *S. aureus* bacteremia with TSS, and suggest that when a patient with TSS presents neurological symptoms, MERS should be considered.

### Availability of data and materials

All the data supporting our findings is contained within the manuscript.

### Consent for publication

Written informed consent was obtained from the patient for publication of this case report and any accompanying images. A copy of the written consent is available for review by the Executive Editor of this journal.

### Ethics approval and consent to participate

Not applicable.

## Abbreviations

ADC: apparent diffusion coefficient
ADEM: acute disseminated encephalomyelitis
CSF: cerebrospinal fluid
DWI: diffusion-weighted imaging
IL: interleukin
MERS: clinically mild encephalitis/encephalopathy with a reversible splenial lesion
MRI: magnetic resonance imaging
MSSA: methicillin-sensitive *S. aureus*
SCC: splenium of the corpus callosum
TNF: tumor necrosis factor
TSS: toxic shock syndrome
